# Influenza and Pertussis Vaccination During Pregnancy: A Systematic Review of Vaccination Rates and Vaccination Determinants

**DOI:** 10.3390/vaccines14040325

**Published:** 2026-04-06

**Authors:** Panagiota Georgia Maltezou, Maria Eleni Papakonstantinou, Eleni Kourkouni, Dimitra Kousi, Christos Hadjichristodoulou, Despoina Briana, Vasiliki Papaevangelou

**Affiliations:** 1 Third Department of Pediatrics, University Hospital Attikon, 124 62 Athens, Greece; mapa7zoo@hotmail.com (M.E.P.); vpapaev@gmail.com (V.P.); 2 Center for Clinical Epidemiology and Outcomes Research (CLEO), 154 51 Athens, Greece; 3 Laboratory of Hygiene and Epidemiology, University of Thessaly, 382 21 Larissa, Greece

**Keywords:** influenza, pertussis, maternal immunization, maternal vaccination, pregnancy, vaccine hesitancy

## Abstract

**Background**: Pertussis and influenza immunization during pregnancy protects both mother and infant through transplacental transfer of antibodies. However, global vaccination coverage among pregnant women remains suboptimal. **Aim**: This systematic review aimed to assess influenza and pertussis vaccination coverage during pregnancy and identify determinants influencing vaccine uptake. **Methods**: A systematic search of MEDLINE, SCOPUS, and grey literature was conducted for studies published between 2000 and 2023. Studies reporting actual vaccination rates for influenza and/or pertussis among pregnant women were included, while those assessing only willingness were excluded. Studies on H1N1 pandemic vaccination in pregnant women were excluded to avoid bias, as awareness levels during the pandemic differed from routine influenza vaccination. Determinants of vaccine acceptance were recorded. Study quality was evaluated using the Newcastle–Ottawa Scale. **Results**: Of 3251 identified records, 78 studies on influenza (N_1_ = 287,124 participants) and 51 on pertussis (N_2_ = 172,801) met inclusion criteria after removing overlapping populations. Most influenza studies (55/78) reported vaccination coverage below 50%. A key determinant of influenza vaccination uptake was physician recommendation, while maternal attitudes, parity, and previous influenza vaccination also had a significant impact. For pertussis, vaccination coverage was primarily driven by physician recommendation, with parity and maternal perceptions of vaccine safety and effectiveness further influencing uptake. Regarding quality assessment, 52.5% of influenza studies and 37.5% of pertussis studies scored above 6 on the Newcastle–Ottawa Scale. **Conclusions**: Maternal vaccination coverage for influenza and pertussis remains inadequate worldwide and is shaped by national strategies, healthcare provider practices, and maternal perceptions. Addressing vaccine hesitancy and improving awareness are essential to increase uptake.

## 1. Introduction

Vaccination during pregnancy is an important public health tool for preventing infections among pregnant women, their fetuses, and infants during the first months of life [1]. Antibodies induced by vaccination during pregnancy are actively transmitted through the placenta to the fetus and by breast milk to the infant. Thus, both the fetus and newborn later are protected from life-threatening diseases during the first months of life, when both cellular and humoral immunity are weak and infant immunization has not yet started [2].

The Advisory Committee on Immunization Practices (ACIP) recommends that all pregnant women should be vaccinated with the inactivated influenza vaccine and pertussis vaccine (combined with diphtheria toxin) [3]. Currently, similar recommendations have been made by the respective vaccination committees in many other countries such as the United Kingdom (since 2010), Belgium, Germany, and Greece [4].

The World Health Organization (WHO) recommendations on maternal immunization have played a pivotal role in shaping influenza and pertussis vaccination coverage over time. Following the prioritization of pregnant women for influenza vaccination in 2012 and the endorsement of maternal pertussis immunization in 2015, several countries adopted or strengthened national recommendations, leading to notable increases in vaccine uptake. Since 2012, studies—especially from high-income countries—have reported markedly higher maternal vaccination coverage, although the magnitude of improvement depends on the strength and implementation of national recommendations [5,6,7,8]. However, the impact of these global guidelines appears to depend on their translation into country-specific policies, as variations in timing, implementation, and integration into antenatal care have resulted in substantial differences in coverage between settings. Overall, both international guidance and national-level recommendations are key determinants of maternal vaccination trends, which have shown a general increase over time [5,6,7,8].

After the COVID-19 pandemic, vaccination against SARS-CoV-2 was added to the maternal immunization schedule, while maternal vaccination against Respiratory Syncytial Virus (RSV) during weeks 32 through 36 of pregnancy has been recently established in many countries [9,10]. The COVID-19 pandemic appears to have had a mixed impact on maternal vaccination coverage. While increased awareness of infectious diseases and vaccination may have contributed to improved uptake in some settings, multiple studies report disruptions in routine healthcare services and reduced access to antenatal care, leading to declines or stagnation in influenza and pertussis vaccination coverage in others. Overall, the evidence suggests that the pandemic introduced both opportunities and setbacks, with its net effect varying across countries depending on healthcare system resilience and public health response [11,12].

Despite scientific evidence confirming the major benefit of vaccinations during pregnancy, epidemiological studies show that pregnant women have poor immunization coverage, even in countries where explicit immunization guidelines for pregnant women exist [1]. This may be attributed to maternal concerns regarding the safety of vaccination during pregnancy, inadequate awareness of vaccine efficacy and the disease burden associated with the respective infections, limited vaccine availability, and insufficient information provided by healthcare professionals [13,14]. Worldwide studies have tried to document pregnant women’s knowledge, attitudes, and practices regarding prenatal vaccination. Most studies show that pregnant women have low vaccination coverage against influenza and pertussis, primarily due to insufficient information from the healthcare provider (HCP) and concerns regarding adverse events [15,16,17,18].

Conducting a systematic review and critical appraisal of relevant data—including vaccination coverage and the determinants of vaccine uptake—can provide valuable insights into the current state of maternal immunization. Such an analysis enables the identification of gaps in coverage, highlights key factors influencing vaccine acceptance, and underscores disparities across different populations or regions. By offering a comprehensive understanding of these issues, the review can support evidence-based decision making and guide the formulation of targeted interventions. Ultimately, these findings may contribute to the development of effective strategies aiming at increasing vaccination coverage and improving maternal and neonatal health outcomes.

## 2. Materials and Methods

The systematic review was conducted according to PRISMA 2020 guidelines [19] (Table S1). The PROSPERO database was searched by the reviewers to make sure that no other research group was working on a systematic review of the subject. PROSPERO received the protocol along with the Prospero registration number, CRD42024605576. Two reviewers (PGM and MEP) independently and blindly conducted a systematic literature search of the MEDLINE and Google scholar databases using an assortment of associated key terms such as “vaccination”, “immunization”, “influenza”, “flu”, “pertussis”, “whooping cough”, “pregnancy”, “pregnant women”. The search query used in Pubmed Medline database was: ((vaccin*[Title/Abstract] OR immuni*[Title/Abstract]) AND (pregnan*[Title/Abstract] OR prenatal*[Title/Abstract])) AND (pertussis [Title/Abstract] OR Bordetella [Title/Abstract] OR “bordetella pertussis” [Title/Abstract] OR flu [Title/Abstract] OR influenza [Title/Abstract]) (File S1). In addition, using a “snowball procedure”, reference lists from all relevant reviews and identified eligible studies, as well as Google Scholar, were manually searched for potentially eligible articles. The grey literature and other relevant articles were identified in this manner. Articles written in English, referring to humans and published between January 2000 and December 2023 and documenting vaccination coverage during pregnancy for either influenza, pertussis or both were included in the analysis. The year 2000 was selected as the lower date limit for this review. The World Health Organization (WHO) and national health agencies began expanding their guidance on maternal immunization, while influenza and pertussis vaccination recommendations for pregnant individuals were significantly revised in 2012 and 2015, respectively [20]. Tracking vaccination rates over the past decade as well could provide a clearer depiction of coverage trends. On the other hand, articles recording influenza or pertussis vaccine uptake during the postpartum period, articles recording solely H1N1 vaccine coverage and articles recording only willingness of vaccination were excluded from the systematic review.

The selection of studies was completed in two stages. First, the titles and/or abstracts of the identified studies were assessed, and those that clearly did not relate to the purpose of the review or did not meet the selection criteria were removed. For further screening of the remaining studies, complete papers were retrieved. Duplicate citations were eliminated after the literature review, and two investigators (PGM and MEP) independently examined the remaining papers to find those that satisfied the pre-established inclusion requirements. Team consensus was used to reach a final decision, in the event of disagreement during the study selection or snowball procedure. If more than one article was discovered with overlapping study populations, the most recent or comprehensive publication was considered eligible. The studies included in the systematic review were assessed using the Newcastle–Ottawa Scale adapted for cross-sectional studies by two reviewers.

### Data Analysis

The study variables of publication year, location, study design, study period, number of mothers or pregnant women involved in the study, and vaccination rate for each vaccine (pertussis and influenza) were extracted from each eligible publication by two reviewers (PGM and MEP). Furthermore, potential factors impacting vaccination rates that were examined by each research team were recorded for all eligible studies. A systematic review regarding vaccination rates and main factors involved in vaccine acceptance was conducted. Moreover, an attempt was made to correlate vaccination rates with different vaccination programs and maternal vaccination recommendations. However, we could not perform a meta-analysis, a fact attributed mainly to the wide variety of statistical analysis and results interpretation methods used in the eligible studies. Missing data in each of the eligible studies were marked as “not available (NA)” and were not included in the systematic review or the qualitative synthesis of the articles.

## 3. Results

**Figure 1:**
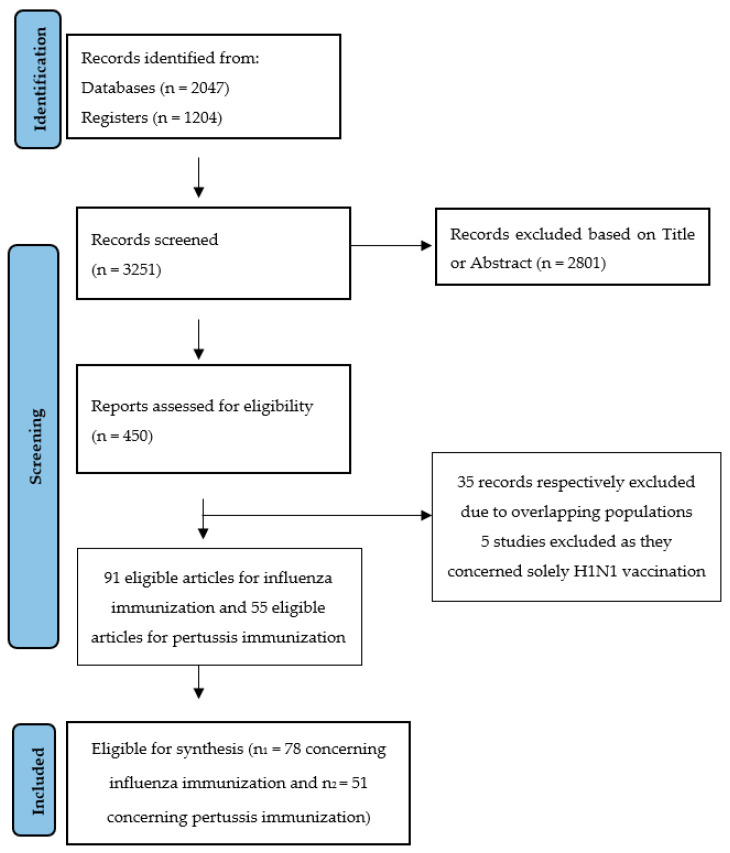
Flowchart of the systematic review [13].

A total of 3251 articles were reviewed for both influenza and pertussis immunization. Out of these, 2801 records were excluded based on Title or Abstract (Figure 1). 

The survey conducted by Specker et al. was excluded as vaccination rate—the primary outcome of our review—was not reported [21]. Kay et al. recorded the flu vaccine uptake peripartum and concluded that 76.9% of the responders had been vaccinated during pregnancy or during the first 2 weeks postpartum. However, since our primary outcome refers to prenatal vaccine uptake, this study was excluded from the final review [22]. Similarly, Bettinger et al. recorded both prenatal and postnatal maternal vaccination rates, namely 45.7% in total, a fact that disqualified their study from being included in our review [23]. After the additional exclusion of influenza and pertussis studies with overlapping populations, 78 and 51 articles, respectively were identified as suitable to be included in the analysis (Figure 1). Risk of bias was assessed using the Newcastle–Ottawa Assessment Scale adapted for cross-sectional studies. Most of the studies reviewed received a score ranging from 6 to 8 points. Points were deducted mainly because of low response rates, lack of multivariable analysis, or due to the use of non-validated screening tools. Studies scoring below 4 points were primarily qualitative studies and interviews that did not quantify and analyze their results using statistics.

### 3.1. Influenza Vaccination During Pregnancy

The detailed characteristics of the 78 eligible studies are depicted in Table 1. The vast majority (77/78, 98.7%) reported findings from cross-sectional studies conducted through either self-completed questionnaires or interviews. Bartolo et al., Descamps et al. and Schlaudecker et al. recorded responses from questionnaires and confirmed data from medical files as well [16,24,25]. Most studies were conducted in the United States (USA) (n = 13), while 18 studies were conducted in low- and middle-income countries. The studies recorded immunization rate from a total of 287,124 pregnant women or new mothers. In addition to vaccination, willingness for immunization was recorded in 14 studies. In 11 studies, separate recordings for seasonal and H1N1 influenza vaccinations were conducted, and the respective data were available. Studies focusing on the H1N1 pandemic and the vaccination of pregnant women with the corresponding vaccine were excluded from the systematic review, as the degree of awareness differed and would introduce bias in the qualitative assessment of the results.

In 37 studies, the vaccination rate against influenza recorded was between 0% to 30%, while 17 surveys concluded that 30–50% of the responders had been vaccinated against flu. In 16 studies, the vaccination rate recorded was 50–70% and 7 studies concluded in a vaccination uptake higher than 70%. Ahluwalia et al. recorded vaccination rates in two different USA regions (Georgia: 18.4% and Rhode Island: 31.9%). The highest vaccination rate, namely 81%, was found in two studies conducted in the USA. The first one was a survey that was carried out by Goldfarb et al. between January and March of 2010, while the second one tracked pregnant women’s vaccination rates from October 2010 to June 2011 [88,90].

Influenza vaccination coverage during pregnancy varied widely across and within country income groups. Higher uptake was generally observed in high-income countries, with rates often exceeding 50% in settings such as Australia, the United States, and the United Kingdom. However, substantial heterogeneity was noted, as some high-income countries (e.g., Italy and France) reported consistently low coverage. Upper- and lower-middle-income countries showed highly variable estimates, ranging from near zero to moderate levels [98]. Overall, these findings suggest that while country income influences vaccination uptake, it does not fully account for the observed differences, indicating a key role for national policies and healthcare system factors.

Vaccination coverage increased over time, with studies conducted before 2010 reporting consistently low uptake. A notable rise was observed following the 2009 H1N1 pandemic and the World Health Organization’s recommendation to prioritize pregnant women for influenza vaccination around 2012. Post-2012 studies, particularly in high-income countries, showed substantially higher coverage rates. Although the introduction of national recommendations was generally associated with improved uptake, the extent of this increase varied across countries, with persistently low coverage in settings where implementation was limited [99].

Most countries introduced recommendations for influenza vaccination during pregnancy between 2009 and 2012, following the H1N1 pandemic and WHO prioritization. In the included studies, higher vaccination coverage was generally observed in settings where national recommendations were implemented earlier and more effectively, whereas countries with delayed or limited adoption showed persistently low uptake. Additionally, higher vaccination rates were observed in studies that had been conducted after the implementation of influenza vaccine uptake during pregnancy.

Post-COVID-19 studies indicate that vaccination uptake during pregnancy varies considerably across countries, without a consistent global trend. High-income countries (e.g., Australia, USA, UK) generally show moderate to high coverage with gradual improvement over time, whereas several European countries maintain lower rates, and some low- and middle-income settings report minimal uptake. The pandemic appears to have had a mixed impact: while increased awareness of infectious diseases may have supported vaccine acceptance in some populations, many studies report persistent or increased vaccine hesitancy among pregnant women, mainly due to safety concerns and mistrust. Overall, COVID-19 did not uniformly increase maternal vaccination coverage but rather highlighted existing disparities among countries.

#### Determinants Affecting Influenza Vaccination

The four main factors that seem to affect vaccine uptake were doctor’s recommendation, maternal positive views regarding influenza vaccine, number of children, and previous influenza immunization. Table 2 depicts the eligible studies that documented these main associations.

Table 3 presents the studies that recorded a statistically significant association between healthcare practiioner’s (HCP’s) recommendation and vaccine uptake.

Expectant mothers’ positive attitude towards influenza vaccination was also correlated with higher vaccination rates in 19 studies (Table 2). Table 4 depicts the statistically significant associations found in eligible studies, as well as the statistical method used.

In 11 studies, parity was examined as a potential determinant of maternal influenza (Table 2). In seven studies, the correlation was proved statistically significant (Table 5). However, the results were contradictory. Namely, in four studies, nulliparity statistically significantly favored influenza vaccination [16,24,39,85], while a statistically significant higher vaccine uptake was clearly recorded in multiparous mothers in two studies [17,64].

Prior vaccination was a main determinant of influenza vaccine uptake in 13 studies. In six studies, the association was proved statistically significant (Table 6).

Regarding Newcastle–Ottawa Scale adapted for cross-sectional studies, the mean score for the eligible studies was 6.18 (Table S2).

### 3.2. Pertussis Vaccination During Pregnancy

Overall, 51 studies on pertussis vaccination during pregnancy were qualified for the systematic review (Table 7). All studies were cross-sectional. In two studies, medical records were used additionally [56,100], while in the study conducted by Kharbanda et al., data regarding study population’s vaccination status was retrieved from the vaccine safety datalink [101]. From the eligible studies, 18 studies were conducted in Europe, while 14 studies were conducted in the USA. Six studies were conducted in low- and middle-income countries. In total, eligible studies included 172,801 women, either pregnant or new mothers, for whom vaccine uptake during pregnancy was recorded. The largest study detected through the literature review was conducted by Butler et al. and published in 2017 (N = 1,147,711) [102]. However, the study had been excluded during the initial steps, due to a high risk of population overlap with other studies conducted in the USA.

The highest vaccination rate, namely 100%, was recorded in the study of Larson Williams et al. conducted in Zambia in 2016 and including 27 women [126]. Notably, in seven studies, authors tried to record the maternal immunization rate as well as the respective willingness of vaccine uptake. In nine studies, the pertussis maternal vaccination rate was less than 30%. In 13 studies, the proportion of vaccinated responders ranged between 30% and 50%, while in 14 studies, the pertussis vaccine uptake was 50–70%. In 14 studies, more than 70% of the participants stated that they had the pertussis vaccine during their pregnancy. In the study conducted by Ben Natan et al. in Israel from January to March 2016, the vaccination rates for native-born women and women derived from the former Soviet Union were 52% and 31%, respectively [108].

Pertussis vaccination coverage during pregnancy varied across income groups. High-income countries generally showed higher uptake, with rates often exceeding 60% in recent studies, although early uptake was lower in some settings (e.g., UK 26% in 2013–2014). Upper-middle-income countries exhibited more variability (Turkey 11–47%, Mexico 74%), while limited data from lower-middle income countries (Zambia) showed very high coverage in targeted programs. These findings indicate that both country income and national policy implementation influence vaccination coverage.

Coverage increased over time in most countries, particularly after the introduction of national recommendations for pertussis vaccination in pregnancy (generally post-2011–2012 following WHO guidance). Strong, well-integrated programs (e.g., Australia, USA, UK) showed substantial improvements, whereas countries with delayed or partial implementation had persistently lower coverage. These results highlight the importance of policy adoption and program integration in achieving high vaccination uptake.

#### Determinants Affecting Pertussis Vaccination

Factors primarily influencing the acceptance of pertussis vaccination were also noted. Doctor’s recommendation, mother’s perception on safety and effectiveness of the vaccine, and parity were main factors that guided maternal immunization (Table 8).

In 20 studies, healthcare provider’s recommendation was declared as an important determinant of pertussis maternal immunization (Table 8). The statistical approach and the respective analysis widely varied. In Table 9, the 14 eligible studies in which statistically significant correlations between HCP’s recommendation and vaccine uptake were recorded are demonstrated.

In six studies, concerns about vaccine safety were stated as an important factor discouraging vaccination (Table 8). However, not all the research teams quantified this association. In three studies, this correlation was proved statistically significant. In five eligible studies, the association between vaccine uptake and pregnant women’s perceptions regarding possible benefits of the antenatal vaccine administration was highlighted. Table 10 and Table 11 depict the statistically significant associations.

Parity was identified as a significant determinant of vaccine acceptance in eight studies (Table 8). In three studies, nulliparous mothers were more likely to get vaccinated when compared to multiparous (Table 12).

Regarding the Newcastle–Ottawa Scale adapted for cross-sectional studies, the mean score of the eligible studies was calculated at 6.13 (Table S3).

## 4. Discussion

Despite the difficulties in synthesizing a vast and diverse body of literature, we have recorded the maternal vaccination rates against influenza and pertussis in many countries worldwide. Additionally, we have estimated the role that several specific beliefs and behaviors play in maternal vaccination. Data from different countries worldwide were combined taking into consideration diverse health systems, immunization strategies, and populations’ perceptions. This systematic review concerns a wide time frame from 2000 till 2023, while the previous respective systematic review published was limited to studies published by November 2018 [127]. Data extracted from CDC recordings has not been used due to the high risk of overlapping populations with studies conducted in the USA. However, the influenza vaccination rates published by the CDC ranged from 40.4% to 50.7% and are equivalent to rates recorded in individual cross-sectional studies from the USA [3,128,129,130,131,132]. It was not possible for a meta-analysis [6] to be performed, since statistical analysis methods had a high heterogeneity and the studies’ methodologies widely differed. Regarding quality assessment, 52.5% of studies concerning influenza vaccine and 37.5% of studies concerning pertussis vaccine scored higher than 6 in the Newcastle–Ottawa Quality Assessment Scale.

In this systematic review, HCPs’ and mothers’ perceptions on vaccine safety and efficacy seem to have a crucial contribution to expectant mothers’ decisions to get vaccinated or not. This finding is also strongly related to each country’s healthcare system and to the HCP who is responsible for pregnant women’s health and follow-up. Only a limited number of studies identified concerns about vaccine adverse events as a key factor influencing maternal vaccination uptake; however, this finding should be interpreted in the context of the historical delay in introducing the Tdap vaccine during pregnancy, which was largely driven by safety concerns for both mothers and infants. Notably, the World Health Organization recommended maternal Tdap vaccination only in 2015, following accumulating evidence demonstrating its safety and effectiveness, while more recent post-COVID-19 studies suggest that vaccine hesitancy related to safety concerns has become increasingly prominent again [6].

Regarding influenza maternal immunization, the lowest rate (0%) was recorded in a study conducted in India during the flu season 2012–2013 [55]. Due to insufficient safety evidence for pregnant Indian women, the influenza vaccine has not yet been made available through immunization programs in India [133]. The highest influenza vaccination rates, reaching 81%, were reported in two U.S. studies conducted immediately following the 2009 H1N1 pandemic—one by Goldfarb et al. from January to March 2010 and another tracking pregnant women from October 2010 to June 2011—which aligns with evidence that the H1N1 pandemic heightened awareness and acceptance of influenza vaccines among pregnant populations [89,90,134]. The respective recommendations from the Advisory Committee on Immunization Practices are strong and prioritize pregnant women as a high-risk group [135,136].

In the present systematic review, the lowest pertussis vaccine uptake recorded in Australia was reported in a study conducted from 2012–2013 [29]. Pregnant women have been gradually enrolled in funded pertussis immunization programs across all Australian states and territories from August 2014 to June 2015, much later than the period the study had recorded. Some of these programs had implemented cocooning strategies as well [137]. The high immunization rate recorded in the study conducted in Zambia may be attributed to the high awareness of infectious diseases in developing countries, the small number of participants, and to the organized funded vaccination strategies imposed in these areas [126]. Our analysis of pertussis vaccination coverage in pregnancy highlights the critical role of national recommendations and program implementation. Countries that introduced formal recommendations earlier (e.g., USA 2011, UK 2012, Belgium 2013–2014, Australia 2015) generally showed higher uptake over time. However, coverage varies widely depending on whether the vaccine was fully integrated into antenatal care and provided free of charge. High-income countries with strong policy support and cost coverage (e.g., Australia, USA, UK, New Zealand) reached coverage often above 70–80%, while countries with partial implementation or out-of-pocket costs (e.g., Turkey, Italy, some regions of Belgium) had lower or more variable coverage. In the study by Ben Natan et al. conducted in Israel (2016), a notable disparity in maternal vaccination coverage was observed, with higher uptake among native-born women compared to those from the former Soviet Union [108]. This difference may reflect variations in health literacy, cultural beliefs, and trust in healthcare systems, as well as potential barriers such as language and access to information. These findings highlight the need for culturally tailored interventions to improve vaccination uptake among migrant populations [108]. These findings underscore that policy recommendation alone is not sufficient; effective integration into healthcare systems and financial accessibility are key determinants of maternal vaccine uptake [138,139,140].

In a previous systematic review and meta-analysis, published in 2020, 21 qualitative studies and 49 quantitative studies were included [127]. Pregnant women who got a recommendation from HCPs had ten to twelve times higher odds of getting vaccinated against influenza or pertussis. In a systematic review of influenza vaccination determinants, which included studies published up to November 2013, the conclusions were similar. Vaccination uptake ranged from 1.7% to 88.4% for seasonal influenza, and from 6.2% to 85.7% for A/H1N1 pandemic influenza. Many pregnant women were unaware that they were at high risk for influenza and its complications during pregnancy. Although HCPs’ recommendations were consistently associated with vaccine uptake, most did not recommend the vaccine to their pregnant clients [141].

Maternal vaccination coverage often decreases with increasing parity, as women with multiple children may face time constraints or perceive lower risk. High- and upper-middle-income countries generally report higher coverage across all parity groups, while low- and lower-middle-income countries show lower uptake, especially among multiparous women. This suggests that access to healthcare, public health recommendations, and socioeconomic factors interact with parity to influence vaccine uptake. Targeted strategies are needed to improve coverage among women with multiple children in lower-income settings [142].

Cyclical influenza and pertussis outbreaks appear to increase vaccine acceptance by raising risk awareness, although this effect is often temporary and depends on sustained public health messaging and access to vaccination services; conversely, in countries such as China and India, where public funding and universal maternal vaccination recommendations have been limited, consistently low vaccination coverage reflects both structural barriers and weaker policy implementation. In addition, regional variations in vaccination coverage observed in the United States and Israel may reflect differences in local acceptance, healthcare access, and prior exposure to outbreaks, suggesting that both epidemiological context and health system factors shape maternal vaccination uptake [11,12,134,138].

In a systematic literature review published by Gkentzi et al., articles regarding pertussis maternal vaccine uptake were reviewed from January 2011 to May 2016, and the main determinants associated with maternal vaccination acceptance were retrieved. Young maternal age, lack of public insurance and premature delivery were factors not favoring vaccine uptake. HCP recommendations proved valuable in this review as well, since in studies conducted in Mexico, Korea and UK, mothers were much more likely to get vaccinated if they had been informed by their HCPs [143].

The main limitations of this systematic review involve the heterogeneity of the eligible studies as far as the study design and the extracted data are concerned. Consequently, the eligible studies did not include the same determinants, and the statistical data analysis was not presented and captured in a single way. It should be noted that different parameters were used for statistical analysis. Some studies used odds ratios to assess the relationship between vaccination rate and specific determinants, while others reported relative risks or prevalence ratios. This resulted in a restrictive descriptive analysis for both qualitative and quantitative data syntheses, making data comparability and data meta-analysis precarious.

## 5. Conclusions

Maternal vaccination rates against influenza and pertussis range worldwide and depend not only on diverse immunization programs and strategies but also on health services and populations’ perceptions. Mothers’ attitudes and knowledge regarding vaccinations and related diseases are crucial regarding maternal vaccination acceptance. Therefore, raising vaccination coverage requires addressing hesitancy and disseminating information about maternal immunization.

## Figures and Tables

**Table 1:** Eligible studies included in the systematic review concerning maternal influenza vaccination.

Country	Publication Year	First Author	Study Period	Study Design	Responders	Age (Years) ^§^	Vaccination Rate	Country’s Recommendation
Australia	2013	Taksdal [26]	November 2012	Cross-Sectional	416	NA	25%	2010
Australia	2013	Wiley [27]	July to November 2011	Cross-Sectional	815	NA	27%	2010
Australia	2013	Maher [28]	November 2011 to December 2012	Cross-Sectional	462	NA	25%	2010
Australia	2014	Collins [29]	2011–2012	Cross-Sectional	17	NA	11.8%	2010
Australia	2015	Hayles [30]	November 2010 to June 2013	Cross-Sectional	2483	NA	39.1%	2010
Australia	2015	Mak [31]	2012–2013	Cross-Sectional	831	NA	36.5%Seasonal	2010
Australia	2015	McCarthy [32]	Each July, 2010 to 2014	Cross-Sectional	1086	NA	42.3% General (29.6% in 2010 to 51.3% In 2014)	2010
Australia	2015	O’ Grady [33]	January To April 2014	Mixed Methods (Cross-Sectional Survey and Yarning Circles (Focus Groups))	53	25 (Range: 17–39)	17%	2010
Australia	2016	Regan [34]	2012–2014 (Every November)	Cross-Sectional	2018	NA	35.3%	2010
Australia	2022	McRae [35]	July to September 2017	Cross-Sectional	642	NA	57.5%	2010
Australia	2018	Lotter [36]	2015	Cross-Sectional	100	26.3 (Range: 18–43)	62%	2010
Australia	2018	Mak [37]	April and October 2015	Cross-Sectional	424	NA	60.6%	2010
Australia	2018	Mohammed [38]	November 2014 And June-2016	Cross-Sectional	180	31.1 (Range: 21–43)	76%	2010
Australia	2021	McHugh [39]	2012–2015	Cross-Sectional	8827	31.8 (Range: 28.5–35.2)	37%	2010
Belgium	2015	Laenen [40]	2015–2018	Cross-Sectional	36,032		14.61%	2010–2011
Brazil	2019	Mendoza-Sassi [41]	January 1 and December 31, 2016	Cross-sectional	2670	NA	53.9%	2010
Canada	2008	Tong [42]	December 2003 to March 2004	Cross-Sectional	185	32.2 (Range: 16–44)	14%	~2007–2009
Canada	2009	Yudin [43]	October to December 2006	Cross-Sectional	58	NA	19%	~2007–2009
Canada	2011	Gracie [44]	November 2009 to March 2010	Prospective Cohort Study	402	31 (19–47)	29.4% Seasonal and H1N1/1.7% Seasonal	~2007–2009
China	2010	Lau [45]	November 2005 To January 2006	Cross-Sectional	568	NA	3.9%	NA
China	2013	Tarrant [46]	February to June 2010	Cross-Sectional	549	NA	4.9% Seasonal/ 2.2% Seasonal and H1N1	NA
China	2017	Song [47]	2012–2014	Cross-Sectional	1673	NA	0%	NA
China	2018	Li [48]	May 2015 to February 2016	Cross-Sectional	108	NA	0%	NA
Czech Republic	2023	Kynčl [49]	September 2020 to August 2021	Cross-Sectional	4617	33 (Range: 18–51)	Less than 2%	~2018–2020
Equador	2021	Erazo [50]	from September 2016 to January 2017	Cross-Sectional	842	NA	36.6%	~2010–2013
France	2016	Loubet [51]	2014–2015	Cross-Sectional and Surveillance System integrated to the European Project Influenzanet	153	34 ± 4.3	26%	~2012
France	2016	Gaudelus [52]	September to December 2014	Cross-Sectional	300	NA	7%	~2012
France	2019	Bartolo [16]	November 2014 to June 2015	Cross-Sectional and Data from Medical Files	2045	NA	35.50%	~2012
France	2020	Descamps [24]	March 2016	Cross-Sectional And Medical Records	11,712	NA	7.4%	~2012
Germany	2014	Boedeker [53]	2013	Cross-Sectional	838	NA	10.90%	2010
Greece	2019	Psarris [54]	April to June 2018	Cross-Sectional	197	NA	16.2%	~2010
India	2014	Koul [55]	October 2012 to April 2013	Cross-Sectional	1000	37 (Range: 18–41)	0%	limited/risk-based
Iran	2012	Honarvar [56]	2010–2011	Cross-Sectional	416	27.06 ± 5.27	6%	ΝA
Iran	2015	Abasi [57]	2013	Cross-sectional	384	NA	1.80%	ΝA
Ireland	2018	Barrett [58]	2016	Cross-Sectional	198	NA	55.1%	2010
Ireland	2018	Hallissey [59]	November 2015 to March 2016	Cross-Sectional	88	31 (Range 18–42)	40%	2010
Ireland	2018	O’Shea [60]	January and June 2016	Cross-Sectional	17	33 (Range: 23–44)	76.40%	2010
Ireland	2018	Ugezu [61]	2018	Cross-Sectional	113	NA	42.50%	2010
Israel	2020	Drezner [62]	December 2017 to July 2018	Cross-Sectional	290	30.5 (19–44)	34.5%	~2009
Italy	2016	Maurici [63]	October to December 2013	Cross-Sectional	309	33.9 ± 4.8	0%	~2012
Italy	2017	Napolitano [15]	December 2015 And February 2016	Cross-Sectional	372	NA	9.7%	~2012
Italy	2018	D’ Alessandro [64]	2017–2018	Cross-Sectional	358	31 ± 5.7 (18–44)	1.4%	~2012
Italy	2022	Scatigna [65]	June to September 2019	Cross-Sectional	251	NA	5.7%	~2012
Italy	2021	Vilca [66]	2018–2019	Cross-Sectional	483	NA	14.9%	~2012
Italy	2023	Ferrari [67]	2019–2022	Cross-Sectional	25,160	NA	18.9%	~2012
Japan	2015	Yamada [68]	March to July 2014	Cross-Sectional	1713	NA	51%	Before 2010
Korea	2021	Kang [69]	October to December 2019	Cross-Sectional	522	NA	63.2%	~2010
Mexico	2014	Varan [70]	2012	Cross-Sectional	387	24 (Range: 15–43)	45%	2011–2012
Nicaragua	2013	Yuet [71]	April to June 2011	Cross-Sectional	2822	NA	1.70%	~2013
Nicaragua	2018	Arriola [72]	June to August 2016	Cross-Sectional	1303	23 y (Range: 13–44)	42%	~2013
Nicaragua	2016	Arriola [73]	October to December 2013	Cross-Sectional	1807	NA	71%	~2013
Saudi Arabia	2017	Mayet [17]	July To August 2013	Cross-Sectional	998	28.4 ± 6.1	18.%	2010–2012
Singapore	2019	Offeddu [74]	September to November 2017	Cross-Sectional	500	NA	9.8%	~2017
South Korea	2014	Kim [75]	2013	Cross-Sectional	218	NA	48.60%	2009–2010
Spain	2018	Castro-Sanchez [76]	November 2015 to May 2016	Cross-sectional	119	32.3 ± 5.5	52%	2005
Spain	2018	Tuells [77]	2014–2015	Cross-Sectional	934	NA	27.9%	2005
Spain	2019	Rodríguez-Blanco [78]	October 2015 to January 2016	Cross-Sectional	683	30.4 ± 5.6	61.6%	2005
Switzerland	2012	Blanchard-Rohner [79]	March 2011	Cross-Sectional	261	NA	18%	~2009–2010
Switzerland	2012	Schindler [80]	March 2011	Cross-Sectional	29	34 (Range: 19–40)	17.2%	~2009–2010
Switzerland	2022	Lumbreras Areta [81]	November 2020 and March to April 2021	Cross-Sectional	950		49.8%	~2009–2010
Turkey	2014	Celikel [82]	2010	Cross-Sectional	198	NA	3% Seasonal	~2010
Turkey	2020	Yakut [83]	November 2015 to May 2016	Cross-Sectional	465	27.8 (Range: 17–42)	19.80%	~2010
UΚ	2018	Wilcox [18]	July 2017 to January 2018	Cross-Sectional	314	NA	38%	2010
UK	2021	Walker [84]	2010/2011–2015/2016	Cross-Sectional	152,132	NA	39.1%	2010
UK	2023	Berendes [85]	April to September 2022	Cross-Sectional	38	NA	50%	2010
USA	2001	Silverman & Greif [86]	January to March 2000	Cross-Sectional	242	NA	8%	2004
USA	2010	Ahluwalia [87]	2006 and 2007	Cross-Sectional	5424	NA	Georgia:18.4% Rhode Island: 31.9%	2004
USA	2011	Ding [88]	April to June 2010	Cross-Sectional	244	NA	32.1%Seasonal	2004
USA	2011	Fisher [89]	November 2009 to May 2010	Cross-Sectional	813	NA	64% Seasonal	2004
USA	2011	Goldfarb [90]	January to March 2010	Cross-Sectional	370	29.8 (15–46)	81% Seasonal and H1N1/7.4% Seasonal	2004
USA	2012	Drees [91]	February to April 2010	Cross-Sectional	307	NA	60% Seasonal	2004
USA/Canada	2012	Gorman [92]	Oct2010 to June2011	Cross-Sectional	199	NA	81%	2004/ ~2007–2009
USA	2013	Eppes [93]	October 2009 to June 2010	Cross-Sectional	88	NA	69% Seasonal	2004
USA	2013	Meharry [94]	May to June 2010	Cross-Sectional	60	33 (Range: 19–40)	51.60%	2004
USA	2015	Henninger [95]	2010–2011	Cross-Sectional	1105	NA	63%	2004
USA	2016	Stark [96]	September 2013 to April 2014 and September 2014 to April 2015	Cross-Sectional	984	NA	71.9%	2004
USA	2018	Strassberg [97]	December 2014 to April 2015	Cross-Sectional	338	NA	70.7%	2004
USA	2019	Schlaudecker [25]	2017	Cross-Sectional and medical files	265	NA	64.9%	2004

^§^ Age of responders is documented as median age, mean age ± standard deviation (SD) or median age and respective age range in brackets according to data available.

**Table 2:** Eligible studies documenting main determinants of influenza vaccine uptake during pregnancy.

First Author, Year	Doctor’s Recommendation	Attitude	Parity	Previous Influenza Vaccination
Ahluwalia, 2010 [87]	✓		✓	✓
Arriola, 2018 [72]	✓		✓	✓
Arriola, 2016 [73]	✓			
Bartolo, 2019 [16]	✓		✓	✓
Blanchard-Rohner, 2012 [79]	✓			
Celikel, 2014 [82]	✓			
D’ Alessandro, 2018 [64]			✓	✓
Descamps, 2020 [24]			✓	
Drees, 2012 [91]	✓			✓
Eppes, 2013 [93]	✓	✓		
Erazo, 2021 [50]	✓	✓		
Ferrari, 2023 [67]	✓			
Gaudelus, 2016 [52]			✓	
Goldfarb, 2011 [90]	✓	✓		
Kang, 2021 [69]	✓			
Lau, 2010 [45]	✓	✓		
Lotter, 2018 [36]	✓			
Loubet, 2016 [51]	✓			
Maher, 2013 [28]	✓			
Mak, 2015 [31]	✓			
Mak, 2018 [37]	✓			
Mayet, 2017 [17]			✓	✓
McCarthy, 2015 [32]	✓			✓
McRae, 2022 [35]	✓	✓	✓	✓
Mohammed, 2018 [38]	✓		✓	✓
Napolitano, 2017 [15]		✓		
Offeddu, 2019 [74]	✓	✓		✓
Scatigna, 2022 [65]	✓	✓		
Schlaudecker, 2019 [25]		✓		
Stark, 2016 [96]		✓		✓
Taksdal, 2013 [26]	✓			
Tarrant, 2013 [46]		✓		
Tong, 2008 [42]	✓	✓		
Varan, 2014 [70]	✓			
Vilca, 2021 [66]	✓	✓		
Wiley, 2013 [27]	✓	✓		
Yamada, 2015 [68]		✓	✓	
Yuet, 2013 [71]	✓	✓		✓

**Table 3:** Statistically significant association between influenza vaccine uptake and doctor’s recommendation ^¶^.

First Author	Year	OR (95% CI)	*p*-Value	RR (95% CI)	PR (95% CI)
Ahluwalia [87]	2010	56.62 (37.43–85.63)	NA	NA	NA
Arriola [72]	2018	74.11 (36.63–149.94)	NA	NA	NA
Arriola [73]	2016	14.22 (10.45–18.40)	NA	NA	NA
Bartolo [16]	2019	18.8 (10.0–35.8)	NA	NA	NA
Blanchard-Rohner [79]	2012	107	NA	NA	NA
Celikel [82]	2014	NA	*p* < 0.001	NA	NA
Drees [91]	2012	NA	NA	3.9 (2.1–7.4)	NA
Eppes [93]	2013	NA	*p* = 0.04	NA	NA
Erazo [50]	2021	NA	NA	NA	Receiving recommendation/not offer → 3.17 (1.57–6.40)/Receiving both recommendation and offer → 15.84 (9.62–26.10) [compared to women who did not receive a recommendation/offer]
Goldfarb [90]	2011	3.06 (1.27–7.38)	NA	NA	NA
Henninger [95]	2015	3.14 (1.99–4.96)	NA	NA	NA
Kang [69]	2021	11.44 (5.46–24.00)	NA	NA	NA
Lau [45]	2010	15.91	*p* < 0.01	NA	NA
Lotter [36]	2018	15.6 (4.9–49.5)	NA	NA	NA
Loubet [51]	2016	41.9 (20.7–84.9)	NA	NA	NA
Maher [28]	2013	41.89 (20.68–84.86)	*p* < 0.001	NA	NA
Mak [31]	2015	11.1 (7.9–15.5)	NA	NA	NA
Mak [37]	2018	influenza recommendation: 4.47 (1.89–10.59) both influenza and pertussis recommendation: 33.3 (15.15–73.38)	*p* < 0.001	NA	NA
McCarthy [32]	2015	NA	*p* < 0.001	NA	NA
McRae [35]	2022	6.06 (3.50, 10.50)	*p* < 0.001	NA	NA
Mohammed [38]	2018	8.0 (3.06–20.9)	*p* < 0.001	NA	NA
Offeddu [74]	2019	NA	NA	NA	PR = 7.11; 95% CI = 3.92–12.90
Scatigna [65]	2022	NA	*p* < 0.001	NA	NA
Taksdal [26]	2013	15.58 (6.055–40.094)	*p* < 0.001	NA	NA
Tong [42]	2008	32.3 (10.4–100)	*p* < 0.0001	NA	NA
Varan [70]	2014	1.95 (1.21–3.15)	NA	NA	NA
Vilca [66]	2021	29.8 (13.1–78.4)	NA	NA	NA
Wiley [27]	2013	20.0 (10.9–36.9)	*p* < 0.01	NA	NA
Yuet [71]	2013	6.30 (3.19–12.46)	NA	NA	NA

^¶^ OR: Odds Ratio; RR: Relative Risk; PR: Prevalence Ratio; 95% CI: 95% Confidence Interval.

**Table 4:** Statistically significant association between influenza vaccine uptake and maternal attitude ^¶^.

First Author	Year	OR (95% CI)	*p*-Value	PR (95% CI)
Eppes [93]	2013	NA	*p* < 0.01	NA
Erazo [50]	2021	NA	NA	1.53 (1.03–2.37)
Goldfarb [90]	2011	OR = 3.92; 95% CI =1.48–10.43	NA	NA
Henninger [95]	2015	participants’ positive views: 2.18 (1.72–2.78)/negative views: 0.36 (0.28–0.460)	NA	NA
Lau [45]	2010	4.97	*p* < 0.01	NA
Napolitano [15]	2017	1.35 (1.14–1.59)	NA	NA
Offeddu [74]	2019	NA	NA	PR = 1.62; 95% CI = 1.30–2.01
Scatigna [65]	2022	NA	*p* < 0.001	NA
Schlaudecker [25]	2019	effective in preventing influenza in themselves (OR 9.0) or their babies (OR 8.1)	NA	NA
Stark [96]	2016	1.708	*p* < 0.01	NA
Tarrant [46]	2013	3.52 (1.37–9.07)	NA	NA
Tong [42]	2008	medium attitude vs. low: 1.62 (1.25–2.1) (*p* = 0.0002)/high attitude vs. low: 4.69 (1.63–13.5) (*p* < 0.0001)	NA	NA
Vilca [66]	2021	2.5 (1.2–6.2)	NA	NA
Wiley [27]	2013	7.6	*p* < 0.01	NA
Yuet [71]	2013	9.98 (3.79–26.24)	NA	NA

^¶^ OR: Odds Ratio; PR: Prevalence Ratio; 95% CI: 95% Confidence Interval.

**Table 5:** Statistically significant association between influenza vaccine uptake and parity ^¶^.

First Author	Year	OR (95% CI)	*p*-Value	RR (95% CI)	PR (95% CI)
Ahluwalia [87]	2010	0.60 (0.40–0.89)	NA	NA	NA
Bartolo [16]	2019	2.5 for nulliparity	NA	NA	NA
D’Alessandro [64]	2018	1.87 for multiparity (1.02–3.41)	NA	NA	NA
Descamps [24]	2020	NA	NA	NA	PR = 2.1 for nulliparity 95% CI = 1.4–3.2
Mayet [17]	2017	NA	*p* < 0.001	NA	NA
Mohammed [38]	2018	0.43 for multiparity; (0.19–0.99)	*p* = 0.048	NA	NA
Yamada [68]	2015	NA	*p* < 0.05	NA	NA

^¶^ OR: Odds Ratio; RR: Relative Risk; PR: Prevalence Ratio; 95% CI: 95% Confidence Interval.

**Table 6:** Statistically significant association between influenza vaccine uptake and previous influenza vaccination ^¶^.

First Author	Year	OR (95% CI)	*p*-Value	RR (95% CI)	PR (95% CI)
Bartolo [16]	2019	4.1 (3.1–5.5)	NA	NA	NA
Drees [91]	2012	NA	NA	Prior influenza vaccination [1.6(1.3–2.0)]/receipt of seasonal influenza vaccination [2.2(1.7–2.9)]	NA
Offeddu [74]	2019	NA	NA	NA	2.51 (1.54–4.11)
Stark [96]	2016	NA	*p* < 0.01	NA	NA
Yuet [71]	2013	2.47 (1.25–4.91)	NA	NA	NA

^¶^ OR: Odds RatioRR: Relative Risk; PR: Prevalence Ratio; 95% CI: 95% Confidence Interval.

**Table 7:** Eligible studies included in the systematic review concerning maternal pertussis vaccination.

Country	Publication Year	First Author	Study Period	Study Design	Responders	Age (Years) ^§^	Vaccination Rate	Country’s Recommendation
Australia	2014	Collins [29]	2011–2012	Cross-Sectional	17	NA	11.8%	2015
Australia	2015	Mak [31]	2012–2013	Cross-Sectional	800	NA	71%	2015
Australia	2018	Lotter [36]	2015	Cross-Sectional	100	26.3 (Range: 18–43)	63%	2015
Australia	2018	Mohammed [38]	November 2014 and June 2016	Cross-Sectional	180	31.1 (Range 21–43)	81%	2015
Australia	2020	Moir [103]	January 2017 to January 2018	Cross-Sectional	1305	NA	82.9%	2015
Belgium	2015	Laenen [40]	December 2013 to February 2014	Cross-Sectional	250	NA	39.2%	2013–2014
Belgium	2016	Maertens [104]	October 2014 and May 2015	Cross-Sectional	823	29.8 ± 4.8	64%	2013–2014
Canada	2022	Li [105]	April to October 2019	Cross-Sectional	946	31 (Range: 28–35)	73.6%	2012–2013
Canada	2022	Gilbert [106]	September 2018 to March 2019	Cross-Sectional	4607	NA	43%	2012–2013
Canada	2023	Wright [107]	2018–2020	Cross-Sectional	243	NA	31.3%	2012–2013
France	2016	Gaudelus [52]	September to December 2014.	Cross-Sectional	300	30.8	7%	2014–2015
Ireland	2018	Hallissey [59]	2015–2016	Cross-Sectional	88	31 (Range: 18–42)	67%	2016
Ireland	2018	O’Shea [60]	2016	Cross-Sectional	17	33 (Range: 23–43)	52.90%	2016
Ireland	2018	Ugezu [61]	2018	Cross-Sectional	113	NA	31%	2016
Israel	2017	Ben Natan [108]	January to March 2016.	Cross-Sectional	200	28.7 ± 4.4. (Range:21–42)	52% of Native-born women compared to 31% of women born in the former Soviet Union	2016
Israel	2020	Drezner [62]	December 2017 to July 2018	Cross-Sectional	290	30.5 ± 5.2 (Range:19–44)	76%	2016
Italy	2018	D’ Alessandro [64]	2017–2018	Cross-Sectional	358	31 ± 5.7 (Range: 18–44)	0%	2019
Italy	2022	Scatigna [65]	June to September 2019	Cross-Sectional	251	NA	16.3%	2019
Italy	2021	Vilca [66]	2018–2019	Cross-Sectional	483	NA	60.9%	2019
Italy	2021	Zambri [109]	October 2019 to January 2021	Cross-Sectional	300	33.3 (Range: 27.3–39.3)	48.3%	2019
Italy	2023	Ferrari [67]	2019–2022	Cross-Sectional	25,160	NA	56.5%	2019
Greece	2019	Psarris [54]	April to June 2018	Cross-Sectional	197	NA	0%	2018
Korea	2021	Kim [75]	October to December 2019	Cross-Sectional	466	NA	67%	2019
Mexico	2014	Varan [70]	2012	Cross-dectional	387	NA	74%	2012
New Zealand	2016	Gauld [110]	2014	Cross-Sectional	37	Range: 18–43	46%	2013
New Zealand	2018	Hill [111]	2013	Cross-Sectional	596	ΝA	74%	2013
New Zealand	2018	Deverall [112]	2017	Clinical audit	111	NA	44%	2013
Spain	2018	Castro-Sanchez [76]	2015–2016	Cross-Sectional	119	32.3 ± 5.5	94%	2015
Switzerland	2022	Lumbreras Areta [81]	November 2020 and March to April 2021	Cross-Sectional	950	NA	86.2%	2020
Turkey	2014	Celikel [82]	March to May 2010	Cross-Sectional	198	NA	47%	2015
Turkey	2020	Yakut [83]	November 2015 to May 2016	Cross-Sectional	465	27.8 (Range: 17–42)	11.20%	2015
UK	2015	Donaldson [113]	2013–2014	Cross-Sectional	200	31(Range: 18–34)	26% vaccinated/ 47.5% willingness	2012
UK	2018	Wilcox [18]	2017–2018	Cross-Sectional	314	NA	56.%	2012
UK	2021	Walker [84]	2012–2015	Cross-Sectional	68,090	NA	67.3%	2012
UK	2023	Berendes [85]	Between April and September 2022	semi-structured interviews and focus group discussion	38	NA	71%	2012
USA	2014	Goldfarb [114]	February to June 2013	Cohort retrospective	1467	NA	81.6%	2011
USA	2014	Housey [115]	November 2011 and February 2013	Cross-Sectional	15,181	21	14.3%	2011
USA	2015	Healy [116]	May 2013 to February 2014	Cross-Sectional	825	30.2 (Range:18–45)	51.6%	2011
USA	2016	Dempsey [117]	2014	Cross-sectional	316	NA	82%	2011
USA	2016	O’Halloran [118]	2013	Cross-Sectional	2958	NA	41.8%	2011
USA	2018	Koerner [119]	October 2013 to September 2014	Cross-Sectional	237	NA	65.8%	2011
USA	2018	Strassberg [97]	2014–2015	Cross-Sectional	338	NA	35.8%	2011
USA	2018	New [120]	2016	Cross-Sectional	66	NA	39%	2011
USA	2018	Khan [121]	March to April 2018	Cross-Sectional	700	NA	54.4%	2011
USA	2019	Kriss [122]	June to July 2014	Cross-Sectional	486	NA	41%	2011
USA	2020	Wales [123]	January to April 2016	Cross-Sectional	400	29.6	65.8%	2011
USA	2019	Shlaudecker [25]	2017	Cross-Sectional	265	NA	89.8%	2011
USA	2020	Badreldin [124]	November 2011–2012, December 2012 and December 2015	Cross-Sectional	2460	NA	44.9% (preguidelines)	2011
USA	2020	Murthy [125]	March to April 2018	Cross-Sectional	700	NA	54.4%	2011
USA	2022	Bernstein [100]	March to August 2017	Cross-Sectional/electronic medical record	1571	NA	43.8%.	2011
Zambia	2018	Larson Williams [126]	2016	Cross-Sectional	50	Median: 27	100%	2016

^§^ Age of responders is documented as median age. mean age ± SD or median age and respective age range in brackets according to data available.

**Table 8:** Principal determinants affecting maternal pertussis immunization.

First Author, Year	Doctor’s Recommendation	Adverse Events	Benefits	Parity
Badreldin, 2020 [124]				✓
Ben Natan, 2017 [108]			✓	
Bernstein, 2022 [100]	✓	✓		
Castro-Sanchez, 2018 [76]	✓			
Celikel, 2014 [82]	✓			
Collins, 2014 [29]	✓			
D’ Alessandro, 2018 [64]				✓
Dempsey, 2016 [117]			✓	
Donaldson, 2015 [113]	✓			
Ferrari, 2023 [67]	✓			✓
Gauld, 2016 [110]	✓	✓		
Goldfarb, 2014 [114]				✓
Healy, 2015 [116]			✓	
Kim, 2021 [75]	✓			
Kriss, 2019 [122]	✓	✓		
Lotter, 2018 [36]	✓			
Maertens, 2016 [104]				✓
Mak, 2015 [31]	✓			
Mohammed, 2018 [38]	✓			✓
Moir, 2020 [103]	✓			✓
Murthy, 2020 [125]		✓		
O’ Halloran, 2016 [118]				✓
Scatigna, 2022 [65]	✓		✓	
Schlaudecker, 2019 [25]	✓			
Strassberg, 2018 [97]	✓			
Varan, 2014 [70]	✓	✓		
Vilca, 2021 [66]	✓	✓	✓	
Wales, 2020 [123]	✓			
Zambri. 2021 [109]	✓			

**Table 9:** Statistically significant association between pertussis vaccine uptake and doctor’s recommendation ^¶^.

First Author	Year	OR (95% CI)	*p*-Value
Bernstein [100]	2022	No HCP’s recommendation: 0.39 (0.23–0.65)	*p* = 0.0003
Celikel [82]	2014	NA	*p* < 0.001
Kim [75]	2021	4.426 (2.114–9.267)	*p* < 0.001
Lotter [36]	2018	13.3 (4.6–38.0)	NA
Mak [31]	2015	33.3 (15.15–73.38)	*p* < 0.001
Mohammed [38]	2018	7.8 (3.3–18.3)	*p* < 0.001
Moir [103]	2020	41.78 (20.03–87.17)	NA
Scatigna [65]	2022	NA	*p* < 0.001
Schlaudecker [25]	2019	5.4	NA
Strassberg [97]	2018	10.231 (3.246–32.247)	NA
Varan [70]	2014	1.95 (1.21–3.15)	NA
Vilca [66]	2021	55.8 (27–127.6)	NA
Wales [123]	2019	NA	*p* = 0.001
Zambri [109]	2021	2.8 (1.4–5.7)	*p* = 0.01

^¶^ OR: Odds Ratio; 95% CI: 95% Confidence Interval.

**Table 10:** Statistically significant association between pertussis vaccine uptake and perceptions about vaccine’s adverse events ^¶^.

First Author	Year	OR (95% CI)	*p*-Value
Bernstein [100]	2022	1.59 (1.12–3.24)	*p* = 0.009
Varan [70]	2014	1.75 (1.16–2.63)	*p* = 0.008
Vilca [66]	2021	2.5 (1.1–6)	NA

^¶^ OR: Odds Ratios of not being vaccinated when fear of vaccine’s adverse events is expressed.

**Table 11:** Statistically significant association between pertussis vaccine uptake and perceptions about vaccine’s benefits ^¶^.

First Author	Year	OR (95% CI)	*p*-Value
Ben Natan [108]	2017	NA	<0.001
Dempsey [117]	2016	NA	0.008
Scatigna [65]	2022	NA	*p* < 0.001
Vilca [60]	2021	3.5 (1.5–8.7)	NA

^¶^ OR: Odds Ratio; 95% CI: 95% Confidence Interval.

**Table 12:** Statistically significant association between pertussis vaccine uptake and parity ^¶^.

First Author	Year	OR (95% CI)	*p*-Value	RR (95% CI)	PR (95% CI)
Badreldin [124]	2020	1.43 (1.05–1.94)	*p* = 0.002	N/A	NA
D’Alessandro [64]	2018	1.87 (1.02–3.41)	*p* < 0.05	NA	NA
Goldfard [114]	2014	0.72 (0.55–0.94)	0.01	NA	NA
Maerteus [104]	2016	NA	*p* = 0.005	NA	NA
Moir [103]	2020	0.38 (0.24–0.6) (second child); 0.18 (0.11–0.31) (subsequent child)	*p* < 0.001	NA	NA
O’Halloran [118]	2016	NA	*p* < 0.05	NA	NA

^¶^ OR: Odds Ratio; RR: Relative Risk; PR: Prevalence Ratio; 95% CI: 95% Confidence Interval.

## Data Availability

Data supporting reported results are available as anonymized databases upon request.

## Supplementary Materials

The following supporting information can be downloaded at: https://www.mdpi.com/article/10.3390/vaccines14040325/s1: Table S1: PRISMA 2020 Checklist; Table S2: Newcastle-Ottawa Assessment Scoring of eligible studies recording influenza maternal immunization; Table S3: Newcastle-Ottawa Assessment Scoring of eligible studies recording pertussis maternal immunization; File S1: Research keywords.

## Abbreviations

The following abbreviations are used in this manuscript:

ACIP	Advisory Committee on Immunization Practices
RSV	Respiratory Syncytial Virus
HCP	Healthcare provider/practitioner
WHO	World Health Organization
SD	Standard Deviation
UK	United Kingdom
USA	United States of America
GP	General Practitioners
OR	Odds Ratio
RR	Relative Risk
PR	Prevalence Ratio
95% CI	95% Confidence Intervals
aOR	Adjusted Odds Ratio
