# Supplementary material for: Influenza and Pertussis Vaccination During Pregnancy: A Systematic Review of Vaccination Rates and Vaccination Determinants
Source: Vaccines (Basel). 2026 Apr 6;14(4):325. doi: 10.3390/vaccines14040325 (PMC13119792; doi:10.3390/vaccines14040325)
Supplement: Supplementary file 1 [file vaccines-14-00325-s001.zip › vaccines-4196890-supplementary/S2_File.pdf]

Supplementary File S2

Search query: ((vaccin*[Title/Abstract] OR immuni*[Title/Abstract]) AND (pregnan*[Title/Abstract] OR prenatal*[Title/Abstract])) AND (pertussis[Title/Abstract] OR bordetella[Title/Abstract] OR "bordetella pertussis"[Title/Abstract] OR flu[Title/Abstract] OR influenza[Title/Abstract])

Limitations: English language, Humans
