# Supplementary material for: Influenza and Pertussis Vaccination During Pregnancy: A Systematic Review of Vaccination Rates and Vaccination Determinants
Source: Vaccines (Basel). 2026 Apr 6;14(4):325. doi: 10.3390/vaccines14040325 (PMC13119792; doi:10.3390/vaccines14040325)
Supplement: Supplementary file 1 [file vaccines-14-00325-s001.zip › vaccines-4196890-supplementary/S3_Table.pdf]

S3_Table: Newcastle-Ottawa Assessment Scoring of eligible studies recording influenza maternal immunization

	**Selection (max 5)**				**Comparability**	**Outcome (max 3)**		**Total Score**
**First Author, Year**	Representativeness of the cases (max1)	Sample size(max1)	Non-response rate(max1)	Ascertainment of the screening/surveillance tool(max2)	The potential confounders were investigated by subgroup analysis or multivariable analysis(max1)	Assessment of the outcome(max2)	Statistical test(max1)	
Abasi, 2015	1	0	1	0	0	0	1	3
Ahluwalia, 2010	1	1	0	1	1	2	1	7
Arriola, 2016	*1*	*1*	*0*	*1*	*0*	*1*	*1*	*7*
Arriola, 2018	2	1	0	1	1	2	1	8
Barrett, 2018	2	0	0	0	1	2	1	6
Bartolo, 2019	2	1	0	0	1	2	1	7
Berendes, 2023	*1*	*0*	*0*	*1*	*0*	*1*	*1*	*6*
Blanchard-Rohner, 2012	2	0	0	1	1	1	1	6
Boedeker, 2014	2	1	0	0	1	2	1	7
Castro-Sanchez, 2012	2	0	0	1	1	2	1	7
Celikel, 2018	1	0	0	1	1	2	1	6
Collins, 2014	1	0	0	1	1	2	1	6
D’ Alessandro, 2018	1	0	0	1	1	2	1	6
Descamps, 2020	1	1	0	1	1	2	1	7
Ding, 2011	1	1	0	1	1	1	1	6
Drees,2012	1	0	0	1	1	2	1	6
Drezner, 2020	2	0	0	1	1	2	1	7
Eppes, 2013	1	0	0	1	0	2	1	5
Erazo, 2021	*1*	*1*	*0*	*1*	*0*	*1*	*1*	*7*
Ferrari, 2023	*1*	*1*	*0*	*1*	*0*	*1*	*1*	*7*
Fisher, 2011	1	1	0	1	1	2	1	7
Gaudelus 2016	1	1	0	1	0	0	0	3

Goldfarb, 2011	2	0	0	1	1	2	1	7
Gorman, 2012	1	0	0	1	1	2	1	6
Gracie, 2011	1	1	0	1	1	2	1	7
Hallissey, 2018	1	0	0	1	0	1	0	3
Hayles, 2015	1	1	0	1	1	2	1	7
Henninger, 2015	1	1	0	1	1	2	1	7
Honarvar, 2012	1	1	0	1	1	2	1	7
Kang, 2021	*1*	*1*	*0*	*1*	*0*	*1*	*1*	*7*
Kim Og Son, 2014	1	0	0	1	1	2	1	6
Koul, 2014	1	1	0	1	1	2	1	7
Kyncl, 2023	1	1	0	1	1	1	1	6
Laenen, 2015	*1*	*1*	*0*	*1*	*0*	*1*	*1*	*6*
Lambreras Areta, 2021	*1*	*1*	*0*	*1*	*0*	*1*	*1*	*7*
Lau, 2010	1	1	0	1	1	2	1	7
Li, 2018	1	0	0	1	0	1	0	3
Lotter, 2018	1	1	1	0	1	2	1	7
Loubet, 2016	1	1	0	1	1	2	1	7
Maher, 2013	1	1	1	0	1	2	1	7
Mak, 2015	1	1	0	1	1	2	1	7
Mak, 2018	1	1	0	1	1	2	1	7
Maurici, 2016	1	0	0	1	0	1	0	4
Mayet, 2017	1	1	0	2	1	2	1	8
Mc Hugh, 2021	1	1	0	1	1	2	1	7
McCarthy, 2015	1	1	0	1	0	1	1	5
McRae, 2022	*1*	*1*	*0*	*1*	*0*	*1*	*1*	*6*
Meharry, 2013	1	0	0	1	0	1	1	4
Mendosa-Sassi	*1*	*1*	*0*	*1*	*0*	*1*	*1*	*7*
Mohammed, 2018	1	0	0	2	1	2	1	7
Napolitano, 2017	1	0	0	1	1	2	1	6
O' Grady, 2015	1	0	0	1	0	1	1	4
Offeddu, 2019	1	1	0	1	1	2	1	7
O'Shea, 2018	1	0	0	2	0	1	1	5
Psarris, 2021	*1*	0	*0*	*1*	*0*	*1*	*1*	*6*
Regan, 2016	1	1	0	1	1	2	1	7

Rodríguez-Blanco, 2019	1	1	0	1	1	2	1	7
Scartigna, 2021	*1*	*0*	*0*	*1*	*0*	*1*	*1*	*6*
Schindler, 2012	1	0	0	1	0	1	0	3
Schaludecker,2019	1	0	0	2	1	2	0	6
Silverman & Greif, 2001	1	0	0	2	1	2	1	7
Song, 2017	1	1	0	0	0	1	0	3
Stark, 2016	1	1	0	1	1	2	1	7
Strassberg, 2018	1	0	0	1	1	1	1	5
Taksdal, 2013	2	1	0	1	1	2	1	8
Tarrant, 2013	1	1	0	1	1	2	1	7
Tong, 2008	1	1	0	1	1	2	1	7
Tuells, 2018	1	1	0	1	1	2	1	7
Ugezu, 2018	1	0	0	1	1	1	1	5
Varan, 2014	1	1	0	1	1	1	1	6
Vilca, 2021	*1*	*1*	*0*	*1*	*1*	*1*	*1*	*6*
Walker, 2021	*1*	*1*	*0*	*1*	*0*	*1*	*1*	*7*
Wilcox, 2018	1	0	0	1	1	1	1	5
Wiley, 2013	1	1	1	1	1	2	1	8
Yakut, 2019	1	1	0	1	1	1	1	6
Yamada, 2015	1	1	0	1	1	2	1	7
Yudin, 2009	1	0	0	1	0	1	0	3
Yuen, 2013	1	1	1	1	1	2	1	8
