# Supplementary material for: Influenza and Pertussis Vaccination During Pregnancy: A Systematic Review of Vaccination Rates and Vaccination Determinants
Source: Vaccines (Basel). 2026 Apr 6;14(4):325. doi: 10.3390/vaccines14040325 (PMC13119792; doi:10.3390/vaccines14040325)
Supplement: Supplementary file 1 [file vaccines-14-00325-s001.zip › vaccines-4196890-supplementary/S4_Table.pdf]

S4_Table. Newcastle-Ottawa Assessment Scoring of eligible studies recording pertussis maternal immunization

First Author, Year	Selection (max 5)				Comparability(max1)	Outcome (max 3)		Total score
	Representativeness of the cases (max1)	Sample size(max1)	Non-response rate(max1)	Ascertainment of the screening/surveillance tool(max2)	The potential confounders were investigated by subgroup analysis or multivariable analysis(max1)	Assessment of the outcome(max2)	Statistical test(max1)	
Badreldin 2020	1	1	0	1	1	2	1	7
Berendes 2023	1	0	0	1	0	1	0	3
Ben Natan 2017	1	0	0	2	1	1	1	6
Bernstein 2022	1	1	0	1	1	2	1	7
Castro-Sanchez 2018	1	0	0	2	1	2	1	7
Celikel 2014	1	0	1	1	1	1	1	6
Collins 2014	1	0	0	2	1	1	1	6
D' Alessandro 2018	1	0	0	2	1	1	1	6
Deverall 2018	1	0	0	2	1	2	1	7
Donaldson 2015	1	0	1	2	1	1	1	7
Drezner 2020	1	0	1	1	1	1	1	6
Ferrari 2023	1	1	0	1	1	2	1	7
Gaudelus, 2016	1	0	0	1	0	1	1	4
Gauld 2016	1	0	0	2	1	1	0	5
Goldfarb 2014	1	1	1	1	1	2	1	8
Hallisey 2018	1	0	0	1	1	1	1	5
Healy 2015	1	1	1	1	1	1	1	7
Hill 2018	1	1	0	1	1	1	1	6
Housey 2014	1	1	1	2	1	2	1	9
Kahn 2016	1	0	0	1	1	1	1	6
Kim 2021	1	1	0	2	1	1	1	7

Koerner 2018	1	0	0	1	1	2	1	6
Kriss 2019	1	1	0	1	1	1	1	6
Laenen 2015	1	1	0	1	0	1	1	6
Larson Williams 2018	1	0	0	2	0	0	0	3
Li 2022	1	1	0	2	1	1	1	7
Lotter 2018	1	1	1	0	1	2	1	7
Lumbreras Areta 2022	1	1	0	1	0	1	1	7
Maertans 2016	1	1	0	1	1	1	1	6
Mak 2015	1	1	1	1	1	1	1	7
Mohammed 2018	1	1	0	1	1	1	1	6
Moir 2020	1	1	0	1	1	1	1	6
Murthy 2020	1	1	1	1	1	1	0	6
New 2018	1	0	0	1	1	2	1	6
O Shea 2018	1	0	0	1	1	1	0	4
O'Halloran 2016	1	1	1	1	1	1	1	7
Psarris 2019	1	0	0	1	1	1	1	5
Scatigna 2021	1	0	0	0	1	1	1	4
Schlaudecker 2019	1	0	0	2	1	1	1	6
Strassberg 2018	1	0	0	1	1	1	1	5
Ugezu 2018	1	0	1	1	1	1	1	6
Vilco 2021	1	1	0	1	1	1	1	6
Wales 2019	1	1	0	1	1	1	1	6
Walker 2021	1	1	0	2	1	2	1	8
Wilcox 2018	1	1	0	1	1	1	1	6
Wright 2023	1	1	0	1	1	1	1	6
Yakut 2019	1	1	1	1	1	1	1	7
Zambri 2021	1	1	1	1	1	1	1	7
